# Rheumatoid Meningoencephalitis: A Feared Condition in the Era of TNF Blockers

**DOI:** 10.1155/2018/4610260

**Published:** 2018-12-16

**Authors:** Fernando Figueroa Rodriguez, Kwon Minkyung, Sruthi Jinna, Sohail Farshad, Francisco Davila

**Affiliations:** Beaumont Health, Department of Medicine, Royal Oak, MI, USA

## Abstract

Central nervous system (CNS) involvement in rheumatoid arthritis (RA) is uncommon, and most of the times, it is secondary to vasculitic processes or extra-articular rheumatoid nodules. Meningeal involvement is extremely rare. In the literature, there are a few case reports, series, and retrospective studies which have demonstrated the association of rheumatoid (aseptic) meningoencephalitis after starting tumor necrosis factor (TNF) inhibitors. We present a case of rheumatoid meningoencephalitis (RME) in a 52-year-old male with a history of RA on adalimumab who presented with headaches associated with motor and sensory deficits, all of which resolved after this diagnosis was achieved and received appropriate therapy with high-dose glucocorticoids. We also present an update with high yield points summarizing clinical features, diagnostic workup, and management of RME. Finally, we post a literature review of relevant CNS rheumatoid disease in patients with and without exposure to TNF inhibitors.

## 1. Case Presentation

A 52-year-old male with a history of rheumatoid arthritis (RA) presented with 3 months of right-sided frontal-parieto-temporal headaches with associated fevers and weight loss. 2 weeks prior to admission, he developed dysarthria and left-sided hemiparesis. He was diagnosed with RA 10 years before and was on adalimumab at the time.

**Figure 1:**
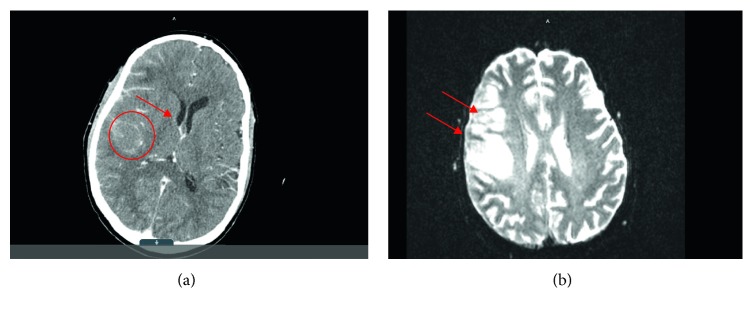
(a) Initial CT scan showing increased diffuse enhancement of right cerebrum as well as ill-defined hypodensity in the posterior right frontal lobe (circle). Slight mass effect is shown upon right lateral ventricle with edema (arrow). (b) MRI brain with gadolinium showing parenchymal and leptomeningeal enhancement over the right frontal and temporal lobes with associated T2/FLAIR signal involving right frontal lobe cortex (arrows), finding suggestive of meningoencephalitis.

**Figure 2:**
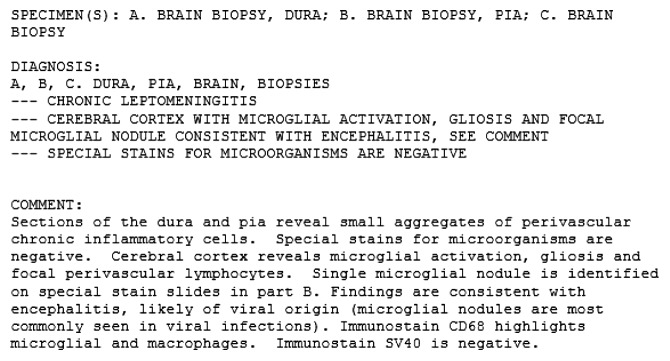
Biopsy report.

**Figure 3:**
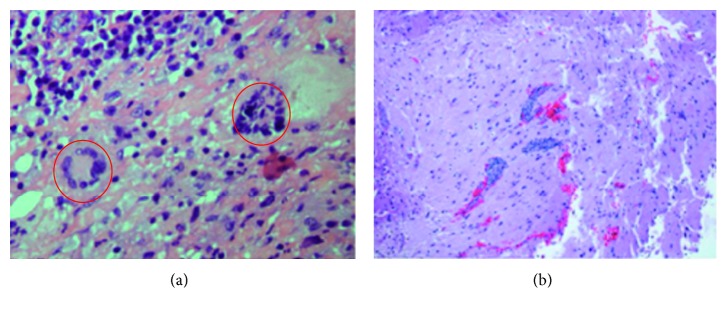
(a) Chronic leptomeningitis with giant cells. (b) Chronic leptomeningitis is demonstrated again; in this cut, parenchymal perivascular lymphocytic infiltrates are appreciated.

Physical exam revealed left-sided facial droop with ipsilateral upper and lower extremities weakness and sensory deficit, and multiple rheumatoid nodules were noted. Laboratory studies revealed leukocytosis with neutrophilia, normocytic anemia, and markedly elevated inflammatory markers. Computerized tomography of the head revealed a right frontal hypodensity (Figure 1). Brain magnetic resonance revealed patchy leptomeningeal enhancement involving right frontal and temporal lobes associated with subcortical edema (Figure 2). Cerebrospinal fluid (CSF) cytology revealed normal glucose with elevated proteins and white blood cells. Cultures from both, CFS and blood, were negative for typical and atypical bacteria, fungi, viruses, and parasites. Brain biopsy was obtained and showed microglial activation and gliosis, suggesting no microorganisms grew from brain tissue culture (Figure 3).

At this point, RME was strongly suspected, so the patient was started on methylprednisolone. He was sent home with long-term steroid taper. On follow-up visits in the office, all signs and symptoms were noted to be resolved.

## 2. Discussion

Central nervous system (CNS) involvement in rheumatoid arthritis (RA) is uncommon, and most of the times, it is secondary to vasculitic processes or extra-articular rheumatoid nodules. Meningeal involvement is extremely rare [1, 2]. Meningoencephalitis after starting TNFs has been described very few times. The incidence of this condition varies ranging from 0.55% to 2% in patients with RA. There are not enough data concerning prognosis, but mortality has been reported as high as 70% [2, 3].

The novelty and importance of our case is fourfold: first of all, RME is an extremely rare condition as detailed above (0.5 to 2% incidence), secondly, it is present in a male, which makes it even rarer. Then, the patient's signs and symptoms resolved with treatment, a situation that is noted to be in less than 30% of the cases. Lastly, this particular patient was on adalimumab, a TNF blocker, which is the center of our discussion and review.

Most CNS symptoms associated with RA are secondary to osseous compression and joint involvement. True inflammation secondary to RA is a rare and potentially treatable condition, whose pathogenesis remains uncertain but can present as a wide spectrum of diseases [1]:

- *Leptomeningitis* (presenting with mental status changes, gait imbalance, memory loss, behavioral changes, seizures, and paresis).
- *Pachymeningitis* (presenting with headaches and cranial neuropathies caused by dural fibrosis).
- *Cerebritis and cerebral vasculitis* are a possibility as well and share some of the symptoms already mentioned. The latter can lead to stroke-like symptoms and thrombosis.

All these conditions are characterized by the absence of organisms in CSF, cultures, and brain biopsy. Spinal fluid analysis might reveal lymphocytic pleocytosis, but it is not always present [4]. An increased level of proteins is characteristic but not specific [5, 6]. New inflammatory markers have been used to aid in diagnosis, specifically rheumatoid factor (RF) in CSF, which is strongly positive in these cases as well as inflammatory cytokines, including TNF alpha, interleukin (IL) 1-beta and IL-6, which are thought to play a central role in the pathogenesis and are often found to be elevated in CSF [7]. MRI with gadolinium contrast is quite helpful by revealing diffuse or patchy enhancement in meninges as well as high intensity lesions in the FLAIR sequence [8]. Ultimately, a brain biopsy is needed for final diagnosis, and any of the following findings are indicative [7, 9]:

- Thickened meninges with nodules and plaques
- Rheumatoid nodules (commonly located in cranial meninges and choroid plexus)
- Nonspecific meningeal inflammation (mainly a mononuclear infiltrate, particularly plasma cells, and less frequently necrosis and multinucleated giant cells)
- Vasculitis (involving brain, spinal cord parenchyma, and meninges)

The differential diagnosis of RME is broad, particularly infectious etiologies due to the use of immunosuppressants, including tuberculosis and fungal infections. Malignancy and drug-induced meningitis are also considerations. The presence of serum anti-CCP antibodies may be helpful in making the diagnosis as well [10–14].

Overall, the preponderance of plasma cells helps distinguish RME from other connective tissue disorders [6–15].

Standard treatment of rheumatoid meningoencephalitis is not established. Medications reported as effective in rheumatoid meningoencephalitis are high-dose corticosteroids, cyclophosphamide, azathioprine, and mycophenolate. It is controversial whether corticosteroids alone are enough to induce remission. The combination of steroids and intermittent cyclophosphamide therapies were effective in some cases [15]. Rituximab alone or in combination with leflunomide has been a successful alternative to high-dose steroids [1, 16]. Some suggests early recognition and treatment with corticosteroid improves survival and mortality, while some reports suggest the opposite [8, 17].

In summary, rheumatoid meningoencephalitis is a poorly understood condition and extremely rare as detailed above. We encourage clinicians to use our case as reference to obtain guidance in diagnosing and managing this condition. We also urge them to report any related cases as this can serve as the basis to better understand it.

## 3. Literature Review

Several of the medications used to treat RA can potentially have a role in the pathogenesis of RME:

- Methotrexate (MTX) can cause rheumatoid nodulosis on the extremities as well as leptomeningitis, probably through induction of adenosine synthesis [1].
- TNF alpha has also been associated with the development of accelerated nodulosis [1, 2]. It has been postulated that TNF blockers may promote development of rheumatoid meningitis via accelerated nodulosis or other unknown mechanisms [3, 18].

For the potential association between TNF blockers and rheumatoid meningoencephalitis, we conducted a literature search from articles published in Medline from 2000 onwards and limited to English. We found 39 cases of rheumatoid meningitis or meningoencephalitis. There were 39 case reports of meningitis/meningoencephgalitis (MME) related to rheumatoid arthritis since 2001; results are as follows (Table 1):

- 64% were female; 36% were male
- Median age was 64 years at the time of MME diagnosis
- Median duration of RA was 10 years at the time of MME diagnosis
- 11 out of 39 patients (28.2%) were exposed to TNF blocker prior to presentation

87% of patients received brain MRI, meanwhile only 64% of the patients underwent brain biopsy for diagnosis. All patients who had brain MRI showed enhancement.

Treatment was attempted with high-dose corticosteroids in most patients (82%).

Cyclophosphamide, azathioprine, or rituximab were also used at a lesser degree. Due to the small number of cases, an association between the treatment agent and outcome was not able to be assessed.

## Figures and Tables

**Table 1:** Overview of patients with rheumatoid meningitis and meningoencephalitis.

	No exposure to TNF blockers	Exposure to TNF blockers
Number of cases reported	28	12
Age, median	66	59
Male (%)	11 (39%)	3 (27%)
Length of illness, median	4.5 years	10 years
Number of patients whose length of illness is greater than 10 years	11 (39%)	6 (55%)
Number of patients with active rheumatoid arthritis, *n* (%)	9 (32%)	6 (55%)

*RA treatment*		
Methotrexate	14 (68%)	9 (82%)
Corticosteroid	11 (39%)	3 (27%)
TNF blocker used, *n* (%)	N/A	Infliximab 6 (50%)
		Adalimumab 4 (33%)
		Etanercept 1 (8.5%)
		Golimumab 1 (8.5%)
Positive brain MRI	100%	8 (73%)

*Meningitis treatment*		
Corticosteroid	23 (82%)	8 (73%)
Cyclophosphamide	7 (25%)	2 (18%)
Other treatments	Azathioprine 1	Azathioprine 1
	MTX 4	Rituximab 1
	TNF blockers 0	Infliximab 1
CSF RF positivity	2	0^*∗*^
CSF WBC, median	36	69
CSF protein, median	67	67
CSF glucose, median	58	48
Positive biopsy or autopsy pathology	19 (68%)	6 (55%)
Good prognosis	19 (68%)	7 (64%)

^*∗*^No author reported CSF RF results in the TNF blocker exposure group.
